# Supplementary material for: Management preferences of orthopedic surgeons in rickets patients in Turkey: Results of a nationwide survey
Source: PLoS One. 2025 Aug 7;20(8):e0329944. doi: 10.1371/journal.pone.0329944 (PMC12331037; doi:10.1371/journal.pone.0329944)
Supplement: S1 Appendix — (DOCX) [file pone.0329944.s001.docx]

**Management Preferences of Orthopedic Surgeons in Rickets Patients in Turkey: Results of a Nationwide Survey**

**1-Experience in years as orthopedic surgeon**

- <5 years
- 5-10 years
- 11-15 years
- 16-20 years
- >20 years

**2-Rate of seeing pediatric patients (0-18 years of age) in daily practice**

- <25%
- 25-50%
- 51-75%
- >75%

**3-Institution**

- Government Hospital
- Education Research/City Hospital
- Government University Hospital
- Foundation/Private University Hospital
- Private Hospital
- Private Practice

**4-Is there a stuff pediatric endocrinologist in your institute?**

- Present
- Absent

**5-Frequency of rickets patient presentation**

- Every day
- >1 in a week
- 1 in a week
- 1 in a month
- <1 in a month

**6-What are the most common complaints of parents suggesting that their child has rickets?**

- Enlargement of the wrists and ankles
- Leg and knee deformities (genu varum. genu valgum)
- Enlargement of the costochondral junction (rachitic rosary)
- Thoracal deformities

**7-What are the concerns that families convey to you about rickets?**

- Gait disorder
- Decreased growth in height
- Having bone fractures
- Deformity in bones

**8-What tests would you order for a child who is suspected of having rickets?**

- I'll do a referral to a pediatric endocrinologist without a test order.
- Calcium
- Phosphorus
- Magnesium
- Alkaline phosphatase
- 25-OH-vit-D3
- Bone survey
- Bone X-rays

**9-What kind of radiological findings would you expect to see in a child with a suspected case of rickets?**

- Metaphyseal enlargement, irregularity and clubbing
- Growth plate enlargement
- Osteopenia in the long bones
- Stress fractures
- Pelvic deformities

**10-Which of the following are types of rickets?**

- Nutritional (calciopenic, phosphopenic)
- Vitamin D-resistant (familial hypophosphatemic)
- Vitamin D-dependent (genetic)

**11-Which option specifies the normal value of 25 OH vitD3?**

- <12 ng/ml <30 nmol/L
- 12-20 ng/ml 30-50 nmol/L
- >20 ng/ml >50 nmol/L
- >100 ng/ml >250 nmol/L

**12-For your patients presenting with complaints/findings of rickets, would you question whether they are regularly taking the vitamin D treatment recommended by their Family Physicians under the age of 1 year?**

- Yes
- No

**13-In patients with 25 OH-VitD3 deficiency, how would you proceed with conservative treatment?**

- I do not start treatment; I recommend consulting a pediatric endocrinologist
- I recommend a diet rich in calcium and phosphorus
- I start oral 25 OH vitD3 drops
- I start oral 25 OH vitD3 ampoules
- I start intramuscular 25 OH vitD3 ampoules
- I start oral 25 OH vitD3 tablets/capsules
- I recommend increasing the frequency of sunbathing

**14-What side-effect(s) are associated with vitamin D preparations if used in an uncontrolled way?**

- Weakness. fatigue
- Anorexia
- Bone pain
- Kidney stone formation
- Cardiac arrhythmia
- Pancreatitis
- Coma
